# Metabolic Pathways Involved in Formation of Spontaneous and Lipopolysaccharide-Induced Neutrophil Extracellular Traps (NETs) Differ in Obesity and Systemic Inflammation

**DOI:** 10.3390/ijms22147718

**Published:** 2021-07-19

**Authors:** Iwona Cichon, Weronika Ortmann, Elzbieta Kolaczkowska

**Affiliations:** Laboratory of Experimental Hematology, Institute of Zoology and Biomedical Research, Jagiellonian University, 30-387 Krakow, Poland; iwona.cichon@doctoral.uj.edu.pl (I.C.); weronika.ortmann@doctoral.uj.edu.pl (W.O.)

**Keywords:** immunometabolism, neutrophil extracellular traps, neutrophils, obesity, GLUT1, glycolysis, PPP pathway, fatty acids, ATP synthase, systemic inflammation

## Abstract

Obesity manifests itself with low-grade chronic inflammation that shapes immune responses during infection. Albeit obese individuals are at risk of higher mortality due to comorbidities, they are better protected from systemic inflammation. Recently, we showed that in the vasculature of obese mice kept on high-fat diet (HFD), neutrophils produce less neutrophil extracellular traps (NETs) than in lean controls (normal diet, ND). NETs are used by neutrophils to counteract severe infection, but they also cause collateral damage. Hardly anything is known about metabolic requirements for their formation, especially in the context of obesity and/or sepsis. Thus, we aimed to study the immunometabolism of NET formation by application of ex vivo neutrophil analyses (Seahorse analyzer, selective inhibitors, confocal imaging) and intravital microscopy. The obtained data show that glycolysis and/or pentose phosphate pathway are involved in NETs release by ND neutrophils in both physiological and inflammatory conditions. In contrast, such cells of septic HFD mice utilize these routes only to spontaneously cast NETs, while after secondary ex vivo activation they exhibit so called “exhausted phenotype”, which manifests itself in diminished NET release despite high glycolytic potential and flexibility to oxidize fatty acids. Moreover, impact of ATP synthase inhibition on NET formation is revealed. Overall, the study shows that the neutrophil potential to cast NETs depends on both the metabolic and inflammatory state of the individual.

## 1. Introduction

Obesity is a chronic progressive disease with remissions and relapses [1]. World Health Organization (WHO) estimates that at least 650 million people worldwide are obese and these numbers steadily increased as they more than doubled between 2000 and 2016 [2]. Obesity is an important driver of the development of multiple associated diseases, and this further increases obesity-associated mortality. In sharp contrast, epidemiological data suggest that obese individuals have higher chances of surviving critical illness conditions such as systemic inflammation [3,4,5]. Be the “obesity paradox in sepsis” true or not, as there are also contradictory reports [6,7], it is commonly accepted that immunological status of an individual is altered during obesity. The low-grade inflammation initiated in the adipose tissue eventually spreads to other metabolic organs, such as the liver, causing long-term deleterious aftereffects [8]. The infiltrating cells are mostly macrophages but also lymphocytes and neutrophils (polymorphonuclear leukocytes, PMN), and their activity is altered in obese individuals [9]. The whole cascade of leukocyte infiltration into adipose tissue is initiated by neutrophils that then recruit macrophages [10]. This is achieved by the release of neutrophil elastase (NE), which facilitates migration though extracellular matrix, but NE also further impairs insulin signaling by promoting insulin receptor substrate 1 (IRS-1) degradation [10]. 

Neutrophils are among first responders of the immune system involved in elimination of infection [11], and NE also plays an important role in this respect, being critical for induction of neutrophil extracellular traps (NETs) [12]. NETs, apart from phagocytosis and degranulation, represent the third weapon utilized by neutrophils but they are two-faced; on the one hand they do catch, immobilize, and (frequently) kill trapped pathogens, but when continually released, overproduced, or not removed efficiently, they cause bystander damage to the endothelium and other tissues [13]. Previously, we studied NET formation in vivo in the vasculature of obese mice, both healthy and with systemic inflammation (endotoxemia), and in either model of obesity—high-fat diet (HFD) or genetic (ob/ob mice)—we observed weaker NET release at the 24-h mark [14]. This phenomenon turned out to be linked to neutrophil-platelet interactions that timewise proceed NET release. Isolated neutrophils of HFD mice casted similar quantities of NETs than those isolated from lean animals (normal diet, ND) 6 h after lipopolysaccharide (LPS) stimulation [14]. To understand metabolic causes behind these phenomena, herein we aimed at comparing metabolic pathways leading to NET release by neutrophils of HFD and ND mice. Not only did we study neutrophils of otherwise healthy obese and lean individuals, but also the cells isolated from mice with ongoing systemic inflammation induced by lipopolysaccharide (endotoxemia).

Metabolically speaking, neutrophils are armed to fuel on glucose. Out of human leukocytes, they have the highest expression of specific glucose transporter proteins GLUT1 and GLUT4, and the second highest of GLUT3 (following monocytes), and they respond to the stimulation by an increased glucose intake [15,16]. Also, murine neutrophils strongly express GLUT1, although not necessarily GLUT3 and 4 [17]. Moreover, they tightly regulate glucose utilization via glycogen cycling to match energy requirements to the immediate demands such as those during inflammation [18]. Additionally, they carry only 5–6 mitochondria on average [19], thus significantly less than other leukocytes or even granulocytes (e.g., eosinophils have 24–36 mitochondria per cell [19]) or other cell types (e.g., hepatocytes contain 1000–2000 mitochondria per cell [20]). In line with this, neutrophil mitochondria are believed to be mostly involved in apoptosis as they hardly participate in adenosine triphosphate (ATP) synthesis and have very low activity of marker enzymes [21]. However, it was also reported that at first mitochondria might provide ATP that initiates PMN activation, and then the cells switch to glycolytic ATP production [22]. Overall, several recent studies revealed that the cells relay not only on glycolysis and pentose phosphate pathway (PPP), but also on Krebs cycle (tricarboxylic acid cycle, TCA), oxidative phosphorylation (OXPHOS), and fatty acid oxidation (FAO) [23,24,25,26].

Immunometabolism is a term coined to describe interactions between metabolic and inflammatory pathways, and studies on metabolic requirements for NET formation and release were initiated. The process is active and requires decondensation of chromatin co-executed by NE and myeloperoxidase (MPO) [27], and then the trap containing a mixture of DNA and proteins of nuclear and granular origin has to be ejected either via membrane rapture (resulting in NETosis) [28] or vital release via vesicles [29]. Thus far, requirement of glucose [30], glycolysis [31,32], and pentose phosphate pathway [33] were confirmed, however, two critical aspects of the above studies have to be underlined, and these are (i) application of a synthetic phorbol myristate acetate (PMA) as a main or exclusive NET inducer, and (ii) neutrophil culturing in full media. Whereas PMA is the most known NET inducer, it is a synthetic compound strongly activating protein kinase C (PKC) controlling phosphorylation of numerous intracellular signaling pathways [34,35,36], whereas clinically-relevant NET inducers do not necessarily activate multiple paths simultaneously. Furthermore, PMA neutrophil stimulation leads to NETosis whereas not all (pato)physiological stimuli cause neutrophil death upon NET release [12,37,38,39]. Moreover, conditions in which NETs are studied are of critical importance, as for example, the presence of serum, proteins, and ions can significantly prone or inhibit their release, either spontaneously or upon endogenous and exogenous inducers [40,41,42].

Metabolic conditions do affect neutrophil functions. For instance, exposure of the cells to increased concentration of low density lipoproteins (LDL) or its oxidized form (oxLDL) affects chemotaxis [43]. Also, glucose excess or deficiency impacts neutrophil chemotactic and phagocytic response to microbials [44,45], superoxide production [46], or NET formation [32,47] with a general tendency of increased glucose concentrations to suppress the cells. However, functional changes in neutrophils in response to different metabolic conditions vary also depending on their duration. For example, a transient glucose elevation may lead to neutrophil activation, however, in prolonged/sustained high glucose environment, neutrophils usually enter a state of immunosuppression [48]. Moreover, metabolic disorders are often associated with either neutropenia or neutrophil dysfunction, namely, impaired chemotaxis and bacterial killing [49]. In obesity (murine HFD-fed model), reduced ex vivo chemotactic response of neutrophils from healthy/uninjured subjects was observed [50], whereas during acute lung injury (ALI), weaker pulmonary neutrophilia was noted in comparison to lean animals [50]. Thus, overall, neutrophils of obese individuals respond differently to inflammatory stimuli than their counterparts of normal/lean subjects.

For the above reasons, we performed analyses of metabolic requirements for NET release by neutrophils of healthy and metabolically challenged mice (with HFD-induced obesity) in a controlled milieu and upon stimulation by bacterial stimulus (LPS). We performed analyses utilizing the Seahorse technology (real time cell metabolic assays) as well as direct ex vivo studies with inhibitors of metabolic pathways. Herein, we report differences in metabolic pathways involved in NET formation by neutrophils of obese and lean mice otherwise healthy or with ongoing endotoxemia. Additionally, we present data on immunometabolism of spontaneous versus LPS-induced NET release.

## 2. Results

### 2.1. Neutrophils of Healthy ND and HFD Mice Depend on Glycolysis in Basal Conditions with Higher Tendency to Glycolytic Functions in Lean Mice

**Figure 1:**
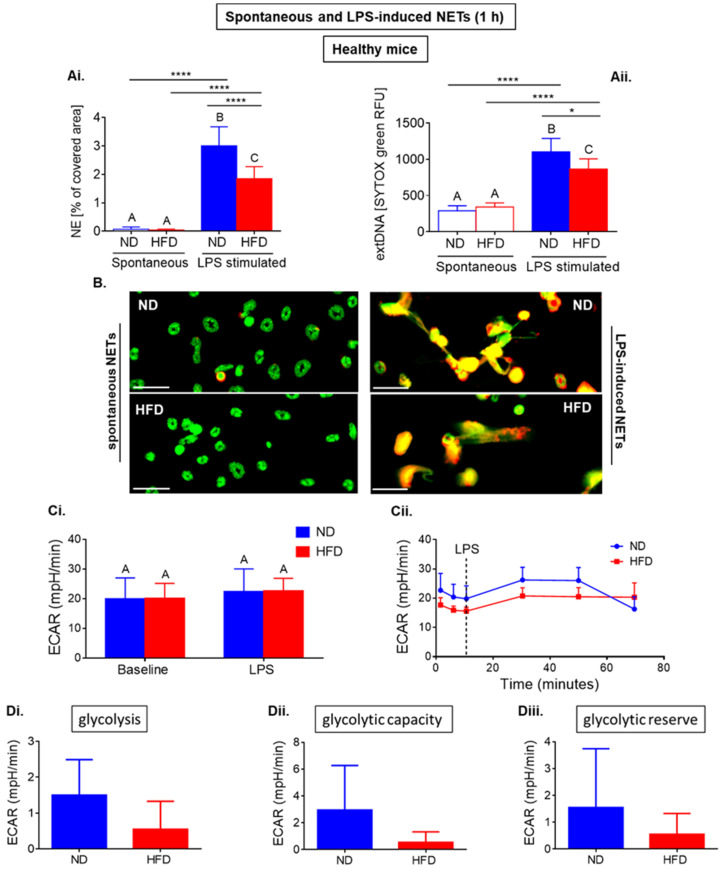
Short-term formation of neutrophil extracellular traps (NETs), and metabolic parameters of neutrophils isolated from healthy mice with obesity induced by high fat diet (HFD) and from their lean controls (ND). Animals were not treated in any way, thus they are described as healthy. NET formation and metabolic parameters were measured within one hour since cell seeding or ex vivo activation with lipopolysaccharide (LPS). NETs were quantified upon staining of neutrophil elastase (NE, ImageJ software) (**Ai**), and (**Aii**) extracellular DNA (extDNA) with SYTOX green; fluorescent signal was quantified using a microplate reader (relative fluorescence units, RFU); (**B**) representative images of NETs are presented for spontaneous NETs of ND and HFD mice and for NETs induced with lipopolysaccharide (LPS). NETs were detected by immunocytochemical counterstaining of neutrophil elastase (NE, red) along with extracellular DNA (extDNA, green). Scale bars indicate: 25 μm. Metabolic parameters were measured with Seahorse analyzer (**C**,**D**). Data quantification of Extracellular Acidification Rate (ECAR) (**Ci**) and a representative Seahorse readout (**Cii**) are presented. (**D**) Glycolysis Stress Test was used to establish glycolysis rate (**Di**), glycolysis capacity (**Dii**), and glycolytic reserve (**Diii**). Asterisks indicate significant differences between groups according to unpaired two-tailed Student’s *t*-test (* *p* ≤ 0.05, **** *p* ≤ 0.0001). Results of one-way ANOVA (post hoc Bonferroni): any two means that do not share same letter are significantly different. Data are shown as mean ± s.d.; n ≥ 3 per group/repetition.

**Figure 2:**
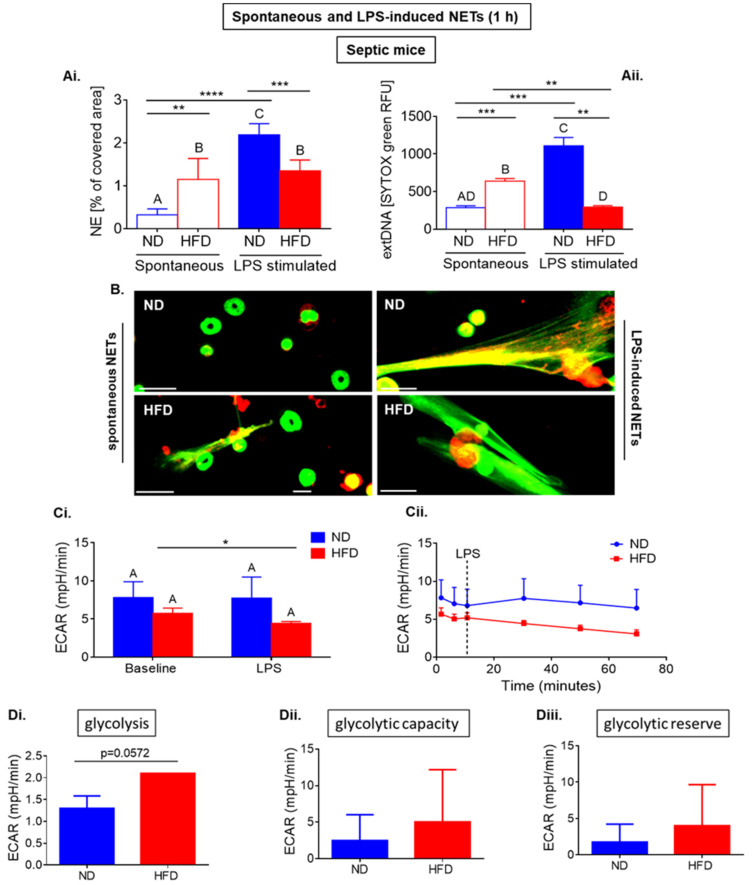
Short-term formation of neutrophil extracellular traps (NETs), and metabolic parameters of neutrophils isolated from septic mice with obesity induced by high fat diet (HFD) and their lean controls (ND). Systemic inflammation/endotoxemia was induced 24 h prior to neutrophil isolation by injecting animals with 1 mg/kg body weight of lipopolysaccharide (LPS). NET formation and metabolic parameters were measured within one hour since cell seeding or ex vivo activation with LPS. NETs were quantified upon staining of neutrophil elastase (NE, ImageJ software) (**Ai**), and (**Aii**) extracellular DNA (extDNA) with SYTOX green; fluorescent signal was quantified using a microplate reader (relative fluorescence units, RFU); (**B**) representative images of NETs are presented for spontaneous NETs of ND and HFD mice and for NETs induced with lipopolysaccharide (LPS). NETs were detected by immunocytochemical counterstaining of neutrophil elastase (NE, red) along with extracellular DNA (extDNA, green). Scale bars indicate: 25 μm. Metabolic parameters were measured with a Seahorse analyzer (**C**,**D**). Data quantification of Extracellular Acidification Rate (ECAR) (**Ci**) and a representative Seahorse readout (**Cii**) are presented. (**D**) Glycolysis Stress Test was used to establish glycolysis rate (**Di**), glycolysis capacity (**Dii**), and glycolytic reserve (**Diii**). Asterisks indicate significant differences between groups according to unpaired two-tailed Student’s *t*-test (* *p* ≤ 0.05, ** *p* ≤ 0.01, *** *p* ≤ 0.001, **** *p* ≤ 0.0001). Results of one-way ANOVA (post hoc Bonferroni): any two means that do not share same letter are significantly different. Data are shown as mean ± s.d.; n ≥ 3 per group/repetition.

To study metabolic pathways triggered just after stimulation with LPS leading to NET formation, we focused on the first 60 min after LPS challenge (data on healthy mice in Figure 1 and data on septic animals in Figure 2). The metabolic analyses were performed with Seahorse analyzer and NETs were studied upon specific staining with the fluorescence and/or confocal microscope.

Firstly, we observed that spontaneous NETs (ejected without any stimulation of neutrophils) are released in the same ratio by neutrophils of ND and HFD mice, whereas one hour after LPS stimulation cells of HFD animals formed less NETs than those of ND mice (as illustrated in Figure 1(Ai,Aii,B)). The same changes were detected for both NET markers, NE, and extracellular DNA (extDNA), and statistical significance was confirmed with both *t*-test and ANOVA.

Within the same time frame, we followed changes in the acidification of extracellular medium resulting from free protons derived mostly from glycolysis (Extracellular Acidification Rate (ECAR)). Cells were placed (in glucose containing assay media) in the instrument and after 12 min of baseline metabolism measurement of ECAR, LPS was injected and reading was continued for 1 h. With this assay, we did not observe differences in response to LPS between ND and HFD neutrophils (as illustrated in Figure 1(Ci) (data from 3 repetitions), Figure 1(Cii) (exemplary Seahorse readout)).

Due to the fact that the ECAR measurement does not allow to exclude protons from other metabolic processes than glycolysis (such as those derived from the Krebs cycle), to measure glycolytic activity more accurately we introduced the Glycolysis Stress Test. In this assay, the cells were seeded in starvation medium (without glucose and pyruvate) stimulated with LPS outside the analyzer and glucose was added during the assay workflow. Glucose deprivation lasted 30 min (the time when the cells adhere to the plate) followed by 18 min measurement of nonglycolytic acidification in glucose free media. After addition of glucose, another 18-min measurement of extracellular acidification was taken which reflects glycolytic rate (glucose-induced increase in ECAR). Subsequently, oligomycin was added to block the ATP synthase and thus inhibit ATP production from mitochondrial respiration, consequently directing neutrophils towards glycolytic metabolism exclusively. The final addition of a glycolysis inhibitor 2-deoxy-D-glucose (2-DG) abolished the overall glycolysis. This strategy allowed to estimate glycolysis capacity (after the ATP synthase blockage; maximum rate of glycolysis) and glycolytic reserve (difference between glycolytic capacity and glycolysis rate; ability to increase glycolytic flux in response to high energetic demands). Although comparison of data did not reach statistical significance, there was a strong tendency to higher glycolytic rate and glycolysis capacity of ND neutrophils (as illustrated in Figure 1(Di,Dii)). We also calculated the glycolytic reserve, and likewise, we observed a tendency to higher glycolytic reserve of neutrophils isolated from lean mice (as illustrated in Figure 1(Diii)). The data on stronger involvement of glycolysis in the activation of neutrophils of ND origin positively correlated with their stronger ability to cast NETs in comparison to that of neutrophils from obese (HFD) mice (as illustrated in Figure 1(Ai,Aii,B)).

### 2.2. Neutrophils of Septic Obese Mice Are Highly Glycolytic but Exhibit “Exhausted Phenotype” after Ex Vivo LPS Stimulation

In contrast to neutrophils collected from healthy obese mice, the cells collected from septic mice and seeded ex vivo for 1 h casted more NETs (“spontaneous NETs”) than that of the ND neutrophils (as illustrated in Figure 2(Ai,Aii,B)). Whereas upon ex vivo LPS stimulation (1-h), not only they did not release more NETs, but in fact they ceased forming NETs (as illustrated in Figure 2(Ai,Aii,B)). The same changes were detected for both NET markers, NE and extDNA, although the difference was more profound in the case of extDNA. This significance of difference was confirmed by both *t*-test and ANOVA.

Importantly, when comparing spontaneous NET formation between healthy and septic mice (as illustrated in Figure 1(Ai,Aii) and Figure 2(Ai,Aii)) we detected stronger NET formation by neutrophils of HFD mice (*p* = 0.0002 extDNA; *p* = 0.0006 for NE) than that of those of lean animals. This suggests that neutrophils of septic obese mice are more prone to release NETs without any stimulation, thus display a proinflammatory phenotype. Additionally, the opposite is observed when the cells are stimulated with LPS (*p* = 0.0015 extDNA; *p* = 0.0268 for NE) which further suggests development of the exhausted phenotype during sepsis in HFD neutrophils. Interestingly, spontaneous NETs seem to be less spread that their LPS-induced counterparts (as illustrated in Figure 2B).

In parallel, we undertook the same metabolic analyses as performed on neutrophils from healthy ND and HFD mice (as illustrated in Figure 1). In septic mice with ongoing endotoxemia, neutrophils of obese (HFD) mice did not respond to LPS in terms of media acidification (ECAR) and the values were significantly lower than in the case of neutrophils obtained from ND animals (as illustrated in Figure 2(Ci) (data from 3 repetitions; difference statistically significant according to *t*-test), Figure 2(Cii) (exemplary Seahorse readout). The response of ND neutrophils to LPS was stronger, but it did not reach statistical significance. Focusing more specifically on glycolysis, we performed Glycolysis Stress Test. Surprisingly, in contrast to data obtained for neutrophils of healthy ND mice, glycolytic rate was higher (*p* = 0.057) in neutrophils from obese animals (HFD) than in that of those from ND ones (as illustrated in Figure 2(Di)) and tended to be higher in case of glycolytic capacity and glycolytic reserve (as illustrated in Figure 2(Dii,Diii)).

### 2.3. Neutrophils of Septic, but Not Healthy, Obese Mice Display Reduced Respiration

**Figure 3:**
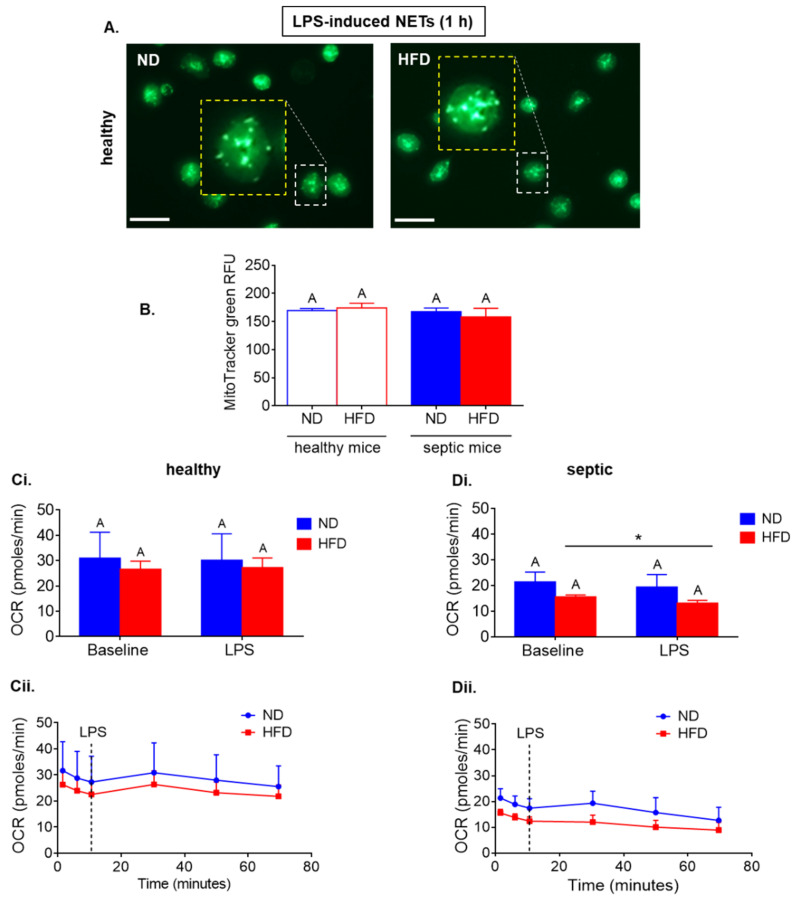
Comparison of mitochondria counts and metabolic activity between neutrophils isolated from obese mice due to high fat diet (HFD) and their lean controls (ND) in a healthy state and with ongoing sepsis. Neutrophils were collected from ND and HFD mice that were either healthy (not treated in any way) or with an ongoing systemic inflammation/endotoxemia induced 24 h prior to neutrophil isolation by injecting animals with 1 mg/kg body weight of lipopolysaccharide (LPS). Metabolic parameters were measured within one hour since cell seeding or ex vivo activation with LPS. Mitochondria were visualized upon staining (MitoTracker green-positive organelles) of neutrophils. Scale bars indicate 20 μm. Representative images for healthy mice are shown (**A**), and data quantification for both healthy and septic mice is shown in (**B**) (RFU, relative fluorescence units). Metabolic parameters were measured with a Seahorse analyzer (**C**,**D**). Data quantification of Oxygen Consumption Rate (OCR) (**Ci**,**Di**) and representative Seahorse readouts (**Cii**,**Dii**) are presented where **C** corresponds to neutrophils isolated from healthy mice, and **D** to their origin from septic mice, respectively. Asterisk indicates significant differences between groups according to unpaired two-tailed Student’s *t*-test (* *p* ≤ 0.05). Results of one-way ANOVA (post hoc Bonferroni): means that share same letter are not significantly different. Data are shown as mean ± s.d.; n ≥ 3 per group/repetition.

As HFD neutrophils so differentially relied on glycolysis depending of their origin (healthy versus endotoxemic mice), we tested what other metabolic pathways they depend on. Firstly, we evaluated if the activity of mitochondria differs between neutrophils of HFD and ND mice, healthy or septic, by two methods of labeling the active organelles. However, both MitoTracker green (as illustrated in Figure 3A,B) and Janus Green staining (data not shown) showed no differences independently of the metabolic state of mice or sepsis presence.

Seahorse analyzes showed that neutrophils isolated from healthy ND and HFD mice did not display differences in oxygen consumption rate (OCR), which is a key metabolic respiration indicator (as illustrated in Figure 3(Ci) (data from 3 repetitions), Figure 3(Cii) (exemplary Seahorse readout)), whereas the HFD cells isolated from septic mice had weaker OCR values in comparison to ND cells (a tendency) and displayed decreased oxygen consumption upon LPS stimulation (as illustrated in Figure 3(Di) (data from 3 repetitions), Figure 3(Dii) (exemplary Seahorse readout)). The significance of difference was revealed by *t*-test. Thus, the pattern was the same as in the case of ECAR (as illustrated in Figure 1 and Figure 2). Additionally, it is worth noting that OCR values increased in healthy mice and septic lean mice within 20 min after LPS stimulation and thereafter gradually decreased, whereas ECAR values were maintained in healthy mice for 40 min (as illustrated in Figure 3(Cii,Dii) vs. Figure 1(Cii)).

### 2.4. Neutrophils of Obese Mice Are More Prone to Switch Their Metabolism between Glucose/Glutamine and Fatty Acid Oxidation Pathways

**Figure 4:**
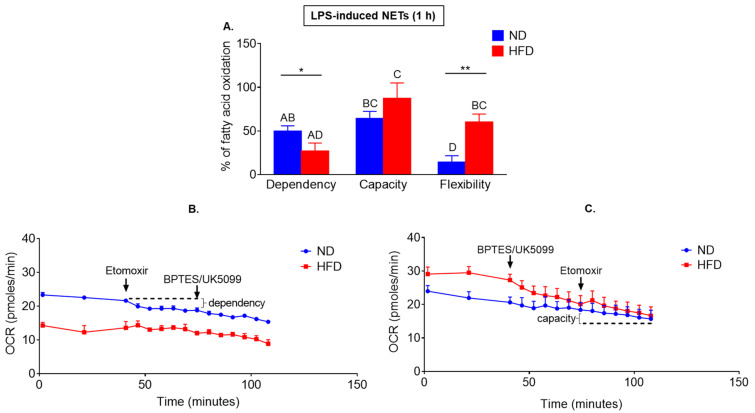
Comparison of fatty acid (FA) oxidation between neutrophils isolated from obese mice due to high fat diet (HFD) and their lean controls (ND). Neutrophils were collected from healthy mice. Oxygen Consumption Rate (OCR) was measured with a Seahorse analyzer applying Mito Fuel Flex Test within two hours since cell activation with lipopolysaccharide (LPS). Two parameters were directly evaluated: neutrophil dependency on FA oxidation (**A**,**B**), cell capacity to oxidize FA (**A**,**C**). Fuel flexibility of the cells was recalculated as difference between their capacity and dependency. Data quantification is shown in (**A**), and representative Seahorse readouts in (**B**,**C**), where (**B**) presents measurement steps and readouts for dependency, and (**C**) for capacity. Asterisks indicate significant differences between groups according to unpaired two-tailed Student’s *t*-test (* *p* ≤ 0.05, ** *p* ≤ 0.01). Results of one-way ANOVA (post hoc Bonferroni): any two means that do not share same letter are significantly different. Data are shown as mean ± s.d.; n ≥ 3 per group/repetition.

Subsequently, we verified involvement of another possible source of energy that is fatty acid (FA) oxidation (as illustrated in Figure 4).

Exogenous fatty acids are taken up by cells, converted to acyl-CoA in the cytosol, and subsequently in a series of reactions, converted to acetyl-CoA in the mitochondria via β–oxidation requiring oxygen consumption and can be directly monitored in real time by means of OCR changes [51,52]. Additionally, neutrophils accumulate lipid droplets and its breakdown provides intracellular source of free fatty acids [24].

Having determined the OCR values, we investigated dependency (cells requirement for fatty acid), capacity (cells ability to metabolize fatty acids) and flexibility (ability to increase the oxidation of fuel of interest to compensate for energy when alternative fuel pathways are inhibited) of neutrophils to oxidize long chain fatty acids. Fatty acid (FA) dependency was tested by first inhibiting fatty acid oxidation (FAO), followed by inhibition of the two alternative fuel oxidation in the mitochondria—glucose and glutamine. FA capacity was tested by first blocking glucose/glutamine oxidation, followed by FAO inhibition. Fuel flexibility was calculated as the difference between capacity and dependency. Whereas neutrophils of obese mice depended less on the process (they were less metabolically active when first fatty acid oxidation was inhibited and later on also the alternative oxidative pathways were blocked) (as illustrated in Figure 4A,B; according to *t*-test), they had the same if not higher (a tendency) capacity to perform it than ND neutrophils (as illustrated in Figure 4A,C). In the latter approach, the opposite treatments were used, i.e., inhibition of the alternative pathways was followed by the inhibition of the target pathway. Thus, overall, neutrophils of obese mice had significantly (*t*-test and ANOVA) higher flexibility in switching between mitochondria-dependent metabolic pathways used.

### 2.5. Upon Longer Incubation, Spontaneously Released NETs Differentially Depend on Glycolysis in HFD and ND Mice

Thus far, we focused on early events occurring just after LPS stimulation of neutrophils and the following NET release. However, the most abundant NET quantities are detected 4–6 h after LPS stimulation [38,53,54,55], thus we aimed to subsequently evaluate later time points. However, when Seahorse analyses were undertaken at these time points (2–6 h), the basic readouts of either ECAR or OCR parameters were below detection level of the apparatus. For this reason, in the following experiments we applied another approach, and namely, we used multiple inhibitors of various metabolic pathways to pretreat neutrophils, and then stimulate them with LPS (or leave them alone to follow spontaneous NET release) and evaluate directly NET formation after 6 h (confocal microscopy).

Firstly, we focused on metabolic requirements for spontaneous NET release as in cultures of untreated neutrophils some NETs are also released. Thus, we kept the cells isolated from HFD and ND mice in culture plates for 6 h and then stained them for NETs; longer incubation (beyond 6 h) was connected with gradual loss of vital cells (data not shown).

**Figure 5:**
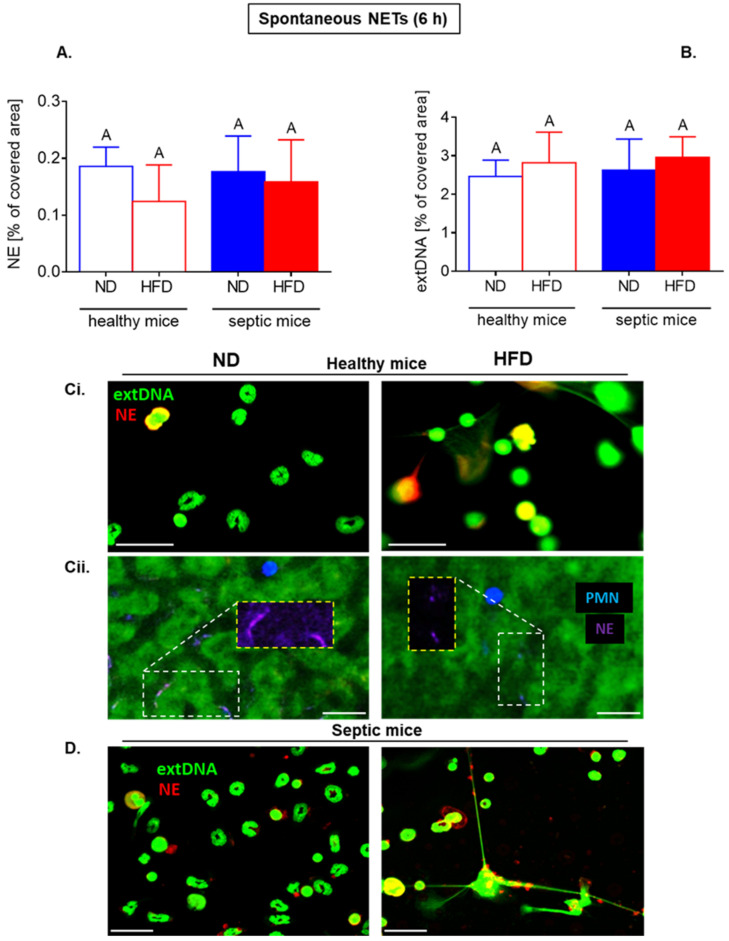
Long-term spontaneous formation of neutrophil extracellular traps (NETs) by neutrophils of mice with obesity induced by high fat diet (HFD) and their lean controls (ND) either ex vivo or in vivo. Studies were performed on either healthy mice (not treated in any way; healthy mice) or with ongoing systemic inflammation/endotoxemia induced 24 h prior to analyses by injecting animals with 1 mg/kg body weight of lipopolysaccharide (LPS; septic mice). Ex vivo NET formation was evaluated 6 h after cell seeding without any further stimulation (**A**–**C**). NET quantification: area [%] covered by neutrophil elastase (NE) (**A**) and extracellular DNA (extDNA) (**B**) Representative images from ex vivo studies: immunocytochemical counterstaining of neutrophil elastase (NE, red) along with extracellular DNA staining (extDNA, green). Scale bar indicates 25 μm (**Ci**,**D**). Additionally, in vivo NET formation in liver vasculature was estimated in healthy untreated mice (**Cii**). On images neutrophil elastase (NE, violet) is visible lining along sinusoids (black ducts) which are localized in between autofluorescent hepatocytes (dim green). White dashed line denotes regions rich in neutrophil elastase. Areas are additionally shown in Cy5 channel (NE signal) only (yellow dashed line). Neutrophils are denoted in blue. Scale bar indicates 25 μm (**Cii**). Results of one-way ANOVA (post hoc Bonferroni): means that share same letter are not significantly different. Unpaired two-tailed Student’s *t*-test did not reveal any differences between groups.

We did not detect any significant differences in NET formation (NE and extDNA) between the two groups of mice independently of their origin from healthy or endotoxemic animals (as illustrated in Figure 5A,B,Ci,D). Interestingly, in vivo, in the vasculature of untreated healthy HFD mice, significantly less spontaneous NETs were imaged than in that of ND ones (as illustrated in Figure 5(Cii)). We should stress here that we studied spontaneous NET release in the vasculature of untreated intact mice, thus no time points apply here as opposed to ex vivo studies.

**Figure 6:**
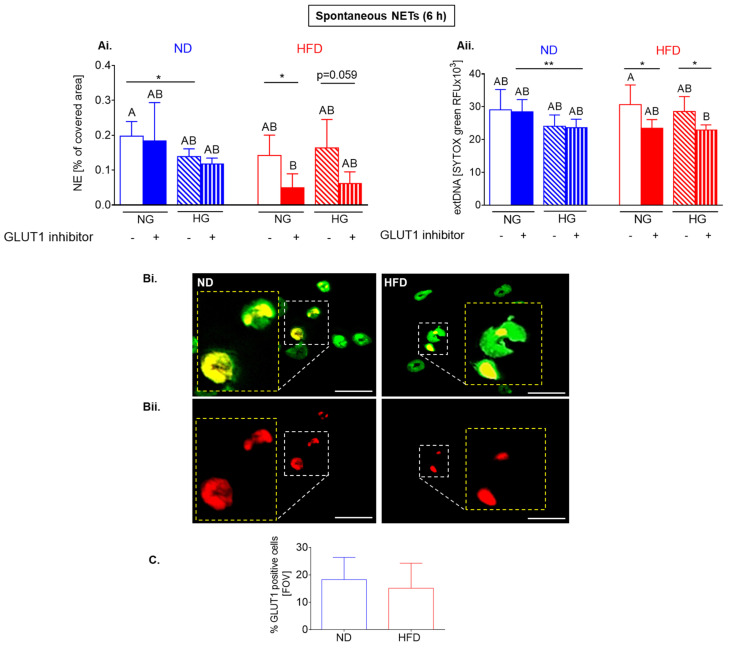
GLUT1-dependent long-term spontaneous formation of neutrophil extracellular traps (NETs) by neutrophils of mice with obesity induced by high fat diet (HFD) and their lean controls (ND). Neutrophils were collected from healthy mice, pretreated with glucose transporter protein 1 (GLUT1) inhibitor, and NET formation was estimated after 6 h. Additionally, some neutrophils were cultured in HBSS supplemented with high glucose (22 mM, HG) or normal glucose (5.5 mM, NG). NETs were quantified upon staining of neutrophil elastase (NE, ImageJ software) (**Ai**), and (**Aii**) extracellular DNA (extDNA) with SYTOX green; fluorescent signal was quantified using a microplate reader (relative fluorescence units, RFU). (**B**) Expression of GLUT1 (red) was estimated in resting neutrophils by immunocytochemistry and is expressed as a percentage of GLUT1-positive cells per field of view (FOV; (**C)**). In (**Bi**), yellow dashed line denotes exemplary cells enlarged by 250% (original location of the cells is marked with a white dashed line). In (**Bii**), only GLUT1-positive signal is shown (red). Scale bar indicates 25 μm ((**B**)—representative images, (**C**)—data quantification). Asterisks indicate significant differences between groups upon unpaired two-tailed Student’s *t*-test (* *p* ≤ 0.05, ** *p* ≤ 0.01). Results of one-way ANOVA (post hoc Bonferroni): any two means that do not share same letter are significantly different. Data are shown as mean ± s.d.; n ≥ 3 per group.

Surprisingly, inhibition of glucose intake (GLUT1 blockage) decreased release of spontaneous NETs only by HFD neutrophils from heathy mice (as illustrated in Figure 6(Ai,Aii)). The opposite data were obtained when LPS was used to stimulate NET release (as illustrated in Figure 8, described in detail below). In ND cells such treatment further diminished NET release by neutrophils incubated in high glucose concentration (as illustrated in Figure 6(Ai,Aii)). High glucose itself did not impact neutrophils of obese mice in any way (as illustrated in Figure 6(Ai,Aii)). The significance of differences was detected by *t*-test.

To verify if the observed differences between ND and HFD neutrophils do not result from various expression of GLUT1, we evaluated its levels but the unstimulated cells from healthy subjects expressed the same quantities of GLUT1 (as illustrated in Figure 6(Bi,Bii,C)).

### 2.6. Upon Longer LPS Stimulation Neutrophils of HFD Mice Release NETs Independently of Glycolysis

**Figure 7:**
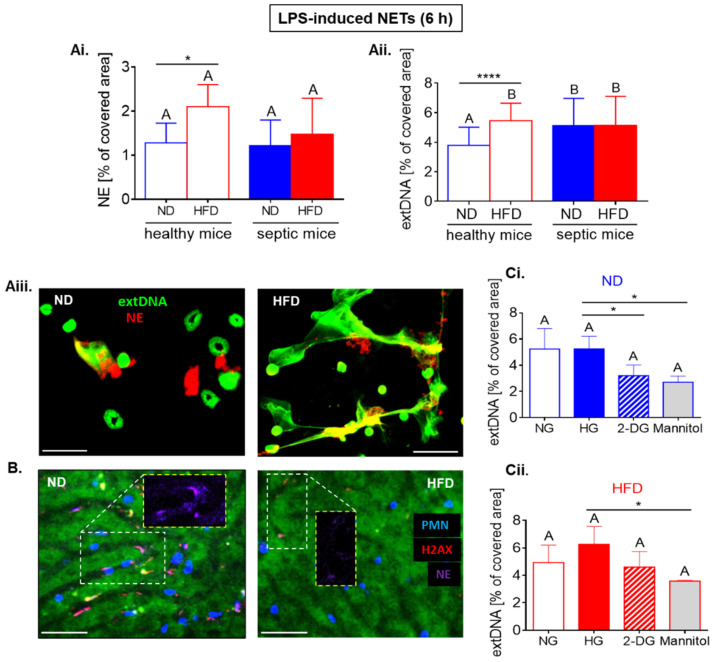
Long-term LPS-induced formation of neutrophil extracellular traps (NETs) by neutrophils of mice with obesity induced by high fat diet (HFD) and their lean controls (ND) either ex vivo or in vivo. Studies were performed on either healthy mice (not treated in any way; healthy mice) or with ongoing systemic inflammation/endotoxemia induced 24 h prior to analyses by injecting animals with 1 mg/kg body weight of lipopolysaccharide (LPS; septic mice). Ex vivo NET formation was evaluated 6 h after cell activation with lipopolysaccharide (LPS; (**Ai**,**Aii**)). NET quantification: area [%] covered by neutrophil elastase (NE) (**A**) and extracellular DNA (extDNA) (**B**) Representative images from ex vivo studies: immunocytochemical counterstaining of neutrophil elastase (NE, red) along with extracellular DNA staining (extDNA, green). Scale bar indicates 25 μm (**Aiii**). Additionally, in vivo NET formation in liver vasculature was estimated at 6 h of endotoxemia (**B**). On images neutrophil elastase (NE, violet) is visible lining along sinusoids (black ducts) which are localized in between autofluorescent hepatocytes (dim green). White dashed line denotes regions rich in neutrophil elastase (violet) and histone H2A.X (red). Areas are additionally shown in Cy5 channel (NE signal) only (yellow dashed line). Neutrophils are denoted in blue. Scale bar indicates 25 μm (**B**). (**C**) In some ex vivo experiments, neutrophils were pretreated prior to LPS stimulation with 2-deoxy-D-glucose (2-DG; **Ci** for ND neutrophils, **Cii** for HFD cells). Additionally, some neutrophils were cultured in HBSS supplemented with high glucose (22 mM, HG) or normal glucose (5.5 mM, NG), or mannitol as osmolarity control (**Ci**,**Cii**). Asterisks indicate significant differences between groups upon unpaired two-tailed Student’s *t*-test (* *p* ≤ 0.05, **** *p* ≤ 0.0001). Results of one-way ANOVA (post hoc Bonferroni): any two means that do not share same letter are significantly different. Data are shown as mean ± s.d.; n ≥ 3 per group.

To follow the same timepoint as for spontaneous NETs (as illustrated in Figure 6), we stimulated neutrophils with LPS for 6 h (as illustrated in Figure 7). Neutrophils collected from septic mice casted the same quantities of NETs (NE and extDNA) independently of their origin, but these collected from healthy obese mice released more traps than those of ND individuals, and this was confirmed by both *t*-test and ANOVA for extDNA and by *t*-test in the case of NE (as illustrated in Figure 7(Ai–Aiii)). This is in sharp contrast to in vivo data, as during endotoxemia HFD, neutrophils released significantly less NETs as verified in the vasculature (also upon 6-h stimulation) (as illustrated in Figure 7B).

**Figure 8:**
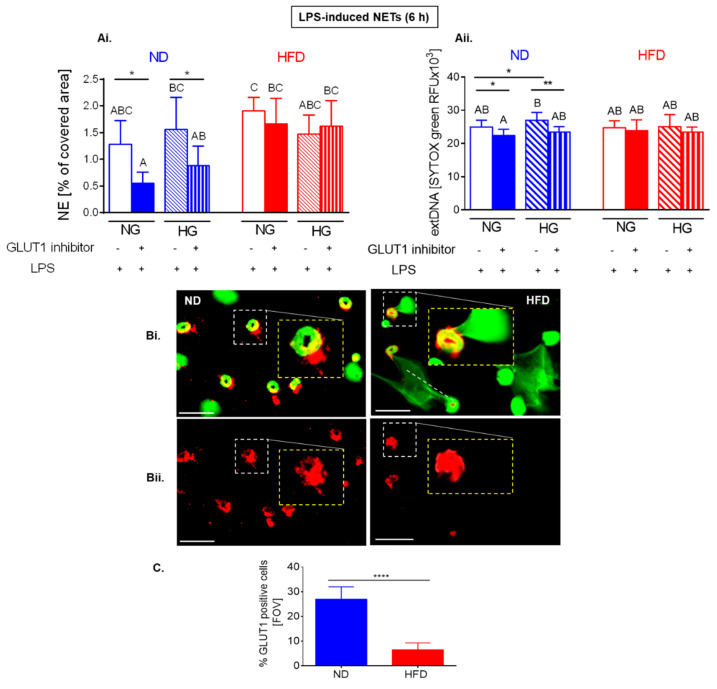
GLUT1-dependent long-term LPS-induced formation of neutrophil extracellular traps (NETs) by neutrophils of mice with obesity induced by high fat diet (HFD) and their lean controls (ND). Neutrophils were collected from healthy mice, and prior to ex vivo studies in which they were stimulated with lipopolysaccharide (LPS) for 6 h, they were pretreated with glucose transporter protein 1 (GLUT1) inhibitor. Additionally, some neutrophils were cultured in HBSS supplemented with high glucose (22 mM, HG) or normal glucose (5.5 mM, NG). NETs were quantified upon staining of neutrophil elastase (NE, ImageJ software) (**Ai**), and (**Aii**) extracellular DNA (extDNA) with SYTOX green; fluorescent signal was quantified using a microplate reader (relative fluorescence units, RFU). (**B**) Expression of GLUT1 (red) was estimated in LPS-stimulated neutrophils by immunocytochemistry and is expressed as a percentage of GLUT1-positive cells per field of view (FOV; **C**). In (**Bi**), yellow dashed line denotes exemplary cells enlarged by 250% (original location of the cells is marked with a white dashed line). In (**Bii**), only GLUT1-positive signal is shown (red). Scale bar indicates 25 μm ((**B**)—representative images, (**C**)—data quantification). Asterisks indicate significant differences between groups upon unpaired two-tailed Student’s *t*-test (* *p* ≤ 0.05, ** *p* ≤ 0.01). Results of one-way ANOVA (post hoc Bonferroni): any two means that do not share same letter are significantly different. Data are shown as mean ± s.d.; n ≥ 3 per group.

Also in the case of LPS-induced NETs, the additional glucose (HG) did not impact on their release by neither ND nor HFD neutrophils (as illustrated in Figure 7(Ci,Cii); significance of differences according to *t*-test). However, inhibition of glucose intake (GLUT1 blockage (as illustrated in Figure 8(Ai,Aii)) or addition of 2-deoxy-D-glucose (2-DG; competitive glycolysis inhibitor; Figure 7(Ci))) decreased NET (NE and extDNA) release only by ND neutrophils. This is in contrast to spontaneous NETs (as illustrated in Figure 6), however, expression of GLUT1 on HFD neutrophils was significantly lowered by 6-h stimulation with LPS (as illustrated in Figure 8(Bi,Bii,C)). The significance of differences was detected by *t*-test.

### 2.7. Upon Longer LPS Stimulation, NET Release Also Depends on PPP but Not Citrate Cycle and Inversely on Oxidative Phosphorylation

**Figure 9:**
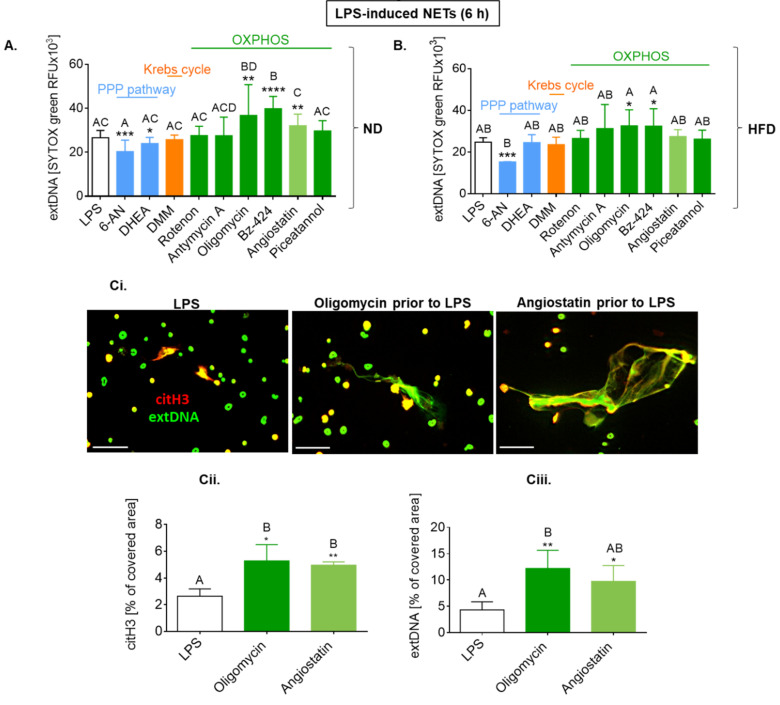
Metabolic pathways involved in long-term LPS-induced formation of neutrophil extracellular traps (NETs) by neutrophils of mice with obesity induced by high fat diet (HFD) and their lean controls (ND). Neutrophils were collected from healthy mice, and prior to ex vivo studies in which they were stimulated with lipopolysaccharide (LPS) for 6 h, they were pretreated with numerous inhibitors. NETs were quantified upon staining of extracellular DNA with SYTOX green and fluorescent signal was measured using a microplate reader in ND (**A**) and HFD (**B**) neutrophil cultures. It is expressed as relative fluorescence units (RFU). Pentose phosphate pathway (PPP) was inhibited with 6-aminonicotinamide (6-AN) and dehydroepiandrosterone (DHEA) (blue columns); Krebs cycle was blocked with dimethyl malonate (DMM, an orange column); OXPHOS was inhibited with a range of inhibitors (rotenone, antimycin A, oligomycin, Bz-423, piceatannol; dark green columns). ATP synthase (mitochondrial and surface) was inhibited with angiostatin (a light green column). (**C**) Effects of oligomycin and angiostatin were recaptured in studies in which NETs were confirmed by immunocytochemical costaining of citrullinated histones H3 (citH3, red) along extracellular DNA (SYTOX green); representative images—(**Ci**), and quantification (area [%] covered by neutrophil elastase (NE, (**Cii**)) and extracellular DNA (extDNA, (**Ciii**))). Scale bar indicates 50 μm. Asterisks indicate significant differences between groups upon unpaired two-tailed Student’s *t*-test (* *p* ≤ 0.05, ** *p* ≤ 0.01, *** *p* ≤ 0.001, **** *p* ≤ 0.0001). Results of one-way ANOVA (post hoc Bonferroni): any two means that do not share same letter are significantly different. Data are shown as mean ± s.d.; n ≥ 3 per group.

To explore what other metabolic pathways might be involved in NET release in HFD mice, we used several inhibitors of other major energy generating pathways that is pentose phosphate pathway (PPP), citrate (Krebs) cycle, and oxidative phosphorylation (OXPHOS) (as illustrated in Figure 9).

Interestingly, the involvement of other metabolic pathways showed similar pattern in NET formation by ND and HFD cells, namely, the process depended on pentose phosphate pathway but did not involve the Krebs cycle and was controlled by OXPHOS (as illustrated in Figure 9(A–Ciii)). The inhibition of the PPP decreased NET formation by HFD cells only upon one inhibitor (6-AN; according to both *t*-test and ANOVA), nevertheless, it is intriguing as GLUT1 blockage and 2-DG should have also inhibited this pathway, yet we did not detect it (as illustrated in Figure 7(Cii) and Figure 8(Ai,Aii)). The most intriguing, however, was the observation that inhibition of ATPases (OXPHOS), either mitochondrial (oligomycin, Bz-424) or surface/mitochondrial (angiostatin), enhanced NET release (as illustrated in Figure 9(A–Ciii)). This difference was confirmed to be significant by both statistical approaches for neutrophils of ND mice, and by *t*-test in the case of the HFD cells.

## 3. Discussion

The current report further confirms the importance of studies on immunometabolism of neutrophils as we show that both intrinsic and environmental factors affect metabolic processes on which neutrophils relay to cast NETs. In particular, we aimed herein to identify engagement of different metabolic fuels and pathways in NET formation by blocking them using specific inhibitors. We focused on multiple variables: (i) origin of neutrophils from either lean (normal weight) mice or obese animals, (ii) healthy or endotoxemic ones, and on (iii) either spontaneous or stimulus-induced NETs, (iv) formed in two different time points.

Time-wise NET release is either studied shortly after stimulation (1–2 h) or several hours post activation (3–6 h). This is partially due to the laboratory settings, but foremost because the early NET formation is rather attributed to the mode of their release in which neutrophils survive the process, whereas the latter is usually connected with neutrophil death (NETosis) [11,41,53,56]. However, in either in vivo or ex vivo studies also spontaneous release of NETs is observed, and whereas it is weaker than upon stimulation, it does occur [40,42]. Ex vivo this is usually attributed to cell handling (isolation procedure, plating/seeding etc.) or response to culture plate materials, especially if their bottom is treated with “tissue culture” (polymers, synthetic or organic) or physically modified (e.g., by plasma gas) to make plastic surface more hydrophilic. Importantly, however, some spontaneously released NETs are also detected in the vasculature of otherwise healthy mice subjected to intravital microscopy (IVM) [57,58]. Moreover, neutrophils were shown to release NETs when exposed to a physical stimulus [59], mechanistic manipulation or hemodynamic force (shear) [28]. Another critical aspect concerns pH/CO_2_ parameters, as for example in acidic conditions, NET formation is reduced due to diminished glycolytic function of neutrophils [60]. NET formation is also associated with energy substrate availability which might limit this active process. For this reason, media formulation (glucose concentration, buffering agents, e.g., HEPES) should be taken into consideration when studying NETs ex vivo. Additionally, one should keep in mind that energetic substrates themselves (e.g., exogenous glucose) can induce NETs without any other immunological stimulation [61]. For this reason, in the current study all neutrophil handling/cultures were performed in basic HBSS with calcium and magnesium, as the addition of serum or even albumins alone inhibits NET formation by murine neutrophils [40]. Additionally, serum might contain DNases which would dissolve NETs immediately upon their release [62]. Moreover, HBSS containing bicarbonate ensures correct control of CO_2_ concentration required for NET formation [63].

Thus far, several studies reported NET-casting dependence on glucose availability. For example, glucose deprivation inhibited NET formation by human cells stimulated with PMA [30] or amyloid fibril [33]. On the other hand, when exogenous glucose (physiological concentration; 4 mM) was added, NET release occurred almost immediately [30]. Also, abnormally high glucose concentration (25 mM) induced NETs ejection from human neutrophils and accordingly, NET markers are elevated in plasma of patients with type 2 diabetes mellitus (T2D) [33]. Consistently, human or mice neutrophils preincubated with 22 mM glucose, and then stimulated with ionomycin/PMA or LPS for 2.5 h, produced more NETs than cells which were incubated with normal glucose concentration (5,5 mM) [32]. However, when we applied the same high glucose concentrations in our studies, we did not see differences in NET release 6 h after LPS by neither ND nor HFD neutrophils. Different LPS concentrations were used in the two studies what might explain this discrepancy. All data reported in the literature thus far were collected from mice kept on a standard diet, and to the best of our knowledge the involvement of glycolysis on NET release was not studied in obese mice and neither were other pathways explored in this respect.

Here, we show that there was a tendency of HFD neutrophils (from healthy, nonseptic mice) activated with LPS to less depend on glycolysis and this correlated with weaker NET release by those cells upon 1-h LPS stimulation. This pattern was altered when neutrophils were isolated from mice subjected to sepsis induced by LPS (endotoxemia). One should keep in mind that during systemic inflammation neutrophils become highly activated, extensively phagocytize and cast NETs as we recently showed by following 24-h kinetics of NET release with IVM [14]. During endotoxemia, HFD neutrophils released less NETs in vivo in the vasculature than ND cells and this was not because of any intrinsic malfunctioning but impaired neutrophil-platelet interactions as revealed for the 6-h time mark [14]. However, we show here that when we isolated neutrophils from septic mice and kept them ex vivo for 1 h, HFD cells were releasing more NETs spontaneously. This could be because the cells were more prone to release NETs without any stimulation (thus display a proinflammatory phenotype) and/or no in vivo restraining signal(s) were present anymore, yet they were primed by endotoxemia (again, displayed a proinflammatory phenotype). In contrast, when we additionally treated the cells with exogenous LPS they stopped casting NETs. These were unexpected, contradictory phenomena. We propose that the latter of them is directly related to the fact that neutrophils received a second hit of LPS (ex vivo), especially that they tend to have higher ratio of glycolysis after LPS stimulation, showing their stronger metabolic capacity to oxidize fuels. However, the cells might have displayed so called an exhausted phenotype disabling further NET release. This phenotype (known previously as immune paralysis of neutrophils) is characteristic for the cells during sepsis [64]. Such exhausted neutrophils show diminished response to following activating stimuli either because they have already secreted their stored granules and NETs [65] or because they become refractory to the immunologic stimulation [64]. We hypothesize that this phenotype was observed only in the case of HFD neutrophils because they are isolated from the restraining in vivo milieu by which we understand, among others, the lack of neutrophil/platelet interactions during endotoxemia [14]; thus, ex vivo, they start to release NETs spontaneously. Correspondingly, stronger release of spontaneous NETs was also observed in the case of human neutrophils isolated from T2D patients [32]. Another possibility being that neutrophils of obese mice are primed by endogenous LPS derived from the gut (metabolic endotoxemia characteristic to obesity) [66], and accordingly, super-low doses of LPS are sufficient to stimulate NETs release [67]. The same applies to increased glucose levels in obesity [68]. On the other hand, pre-exposure to LPS might reduce the sensitivity of myeloid cells to a second LPS challenge, a phenomenon referred to as LPS tolerance or endotoxin tolerance [69]. At this point we do not know which of the two phenomena occurred in our model—the exhaustion being defined as a state of functional hyporesponsiveness or tolerance as a state of functional unresponsiveness [70]. However, when we isolated neutrophils from healthy obese mice, they formed spontaneous NETs in the same ratio as ND cells implying that this effect should rather be attributed to provoked endotoxemia (exogenous LPS), and thus rather exhaustion. Moreover, to make sure that the differential response of lean and obese neutrophils does not result from dissimilar expression of TLR4, we checked it, but no differences were detected between the groups (as illustrated in Supplementary Figure S1). Although herein we focus on metabolic differences between neutrophils of lean and obese mice, we cannot exclude a possibility that they are due to, or linked to, existence of different neutrophil subtypes in these mice. This, however, requires further studies.

We observed that glycolysis was more active in septic HFD neutrophils at 1 h of LPS stimulation, whereas their oxidative phosphorylation was weaker despite unchanged overall mitochondrial activity, even though neutrophils are mainly glycolytic and contain few mitochondria [21,71]. Seahorse analyses were able to detect reduced respiration in neutrophils from septic obese mice. OCR response, monitored concurrently with ECAR, serves as an indicator of whether glucose is also catabolized through mitochondrial respiration. Less oxygen consumed by neutrophils from septic HFD mice suggests that they produce less ATP via electron transport chain (ETC); however, at the same time, they are more glycolytic (glycolytic stress test). Importantly, metabolic changes within the mitochondria have indispensable role in neutrophil activation, as ATP production by complex V of ETC drives neutrophils via purinergic signaling mechanism [22]. If so, it may also explain reduced NET formation by neutrophils from obese individuals at 1 h. It should be underlined that OCR values increased within 20 min after LPS stimulation in both groups of mice, and thereafter gradually decreased, unlike in the case of ECAR values. This suggests that metabolic changes within mitochondria are initiated very rapidly and precede the resulting functional processes, such as NET release, which occur later. Similar observations on neutrophil phagocytosis and degranulation were reported previously revealing that the burst of ATP release generated by the mitochondria fuels the first phase of purinergic signaling that boosts Ca^2+^ signaling, amplifies mitochondrial ATP production, and initiates functional neutrophil responses (phagocytosis or degranulation). Next to these changes within the mitochondria, neutrophils utilize glucose for glycolytic ATP production in the cytoplasm, which in turn fuels a second round of purinergic signaling that sustains Ca^2+^ signaling [22]. Despite the undermined role of mitochondria in neutrophil biology, in some circumstances, NET casting might relay on mitochondrial ROS (mtROS) [72]. Some reports indicate that NOX-independent NETs formation (ionomycin-induced) depends on mitochondrial ROS (mtROS) [73], as some other processes [74], although also contradictory data were reported on NETs [33,72].

Interesting also are the conclusions from analyses of fatty acid oxidation (FAO) as the energy source. HFD neutrophils depend less than neutrophils from ND mice on fatty acids oxidation although they have high capacity to utilize this fuel. Most importantly, however, they display high flexibility in switching between mitochondrial metabolic pathways used—aerobic glycolysis/glutaminolysis and FAO, to meet energy demands.

We also wanted to verify if the metabolic pathways used by neutrophils change over time when their incubation or stimulation is prolonged. Seahorse analyses undertaken beyond two hours were unfeasible as ECAR and OCR parameters were far below detection levels of the apparatus (the recommended range of the XFp). Therefore, in the following studies, we focused on direct effects of specific inhibitors of metabolic pathways on NET formation. The fact that ECAR and OCR were so low several hours after neutrophil seeding/stimulation, yet we could clearly see the effects of some of the inhibitors added prior to LPS, could be because neutrophil activation, including NET induction, is initiated shortly after it, whereas actual NET release is delayed in time [30]. This issue was discussed above. In fact, NET discharge (their physical release from the cell) does require time from the stimulation till the extracellular release of the traps [75]. This was interestingly explained from the metabolic point of view in a study when neutrophils were exposed to PMA in glucose-free medium for 3 h. In such conditions, they lost their characteristic polymorphic nuclei (chromatin decondensation occurred) but they did not release NETs extracellularly. Once glucose was added, the NET release took place within minutes. This study suggests that chromatin decondensation depends on endogenous glucose but the excretion itself mostly relays on exogenous glucose [30].

When it comes to the release of spontaneous NETs at 6 h, neutrophils of ND and HFD mice were forming the same quantities regardless of their origin from healthy or septic mice. Thus, there was no more increased release by cells originating from septic mice. This suggests that indeed, neutrophils from either group of mice have the same potential to cast NETs once the conditions are normalized for sustained period. In contrast, 6-h ex vivo LPS stimulation reversed the phenotype observed at 1 h, as at that time neutrophils collected from healthy mice were in fact releasing more NETs. Also, the cells collected from septic animals now started releasing the traps and they were doing it as efficiently as ND neutrophils. This could be due to reversed exhaustion, a phenomenon originally described in T cells. Prolonged antigen exposure due to viral infection [76] or cancer [77] leads to the dysfunctionality of lymphocytes, but when e.g., pathogen is eliminated, they can be, at least partially, reinvigorated [78]. Mechanisms of this process include PD-1 molecule [79] which expression is also increased on exhausted neutrophils [64] and augments during infection [80], thus the neutrophil exhaustion might also be possibly reversible. In the case of neutrophils, however, it would indicate that prior to exhaustion the cells did not release NETs, and instead they were irresponsive to the original stimulus and did not become exhausted because they already casted NETs.

The increased capacity of HFD neutrophils collected from healthy mice to cast NETs upon LPS stimulation after several hours inversely correlated with their GLUT1 expression. It was shown previously in mice that LPS stimulation does not increase GLUT1 expression by neutrophils, but rather, it induces its translocation from the cytoplasm to the cell surface along with a dose-dependent increase in [^3^H]DG uptake [17]. In line with this, in healthy mice we observed the same expression of GLUT1 in unstimulated neutrophils from ND and HFD mice and the signal was mostly localized in the cytoplasm rather than in the plasma membrane. In contrast, in LPS-stimulated neutrophils, although to a lower extent in HFD cells, the expression was observed mostly on the cell surface, which is in agreement with previously published observations [17]. The reason behind profoundly decreased expression of GLUT1 in neutrophils of obese healthy mice is unclear. There are reports on altered GLUT1 expression in various cell types of obese individuals, e.g., in the adipose tissue their expression is higher [81] whereas in splenic lymphocytes [82] or brain endothelial cells [83] lower than in lean subjects. Moreover, GLUT1 expression is reduced in skeletal muscle cells in T2D characterized by increased glucose levels [84]. However, at the resting state neutrophils from our obese mice expressed GLUT1 similarly to those from control animals, and only HFD cells activated with LPS displayed lower expression of that transporter. If so, this might indicate that the process of GLUT1 surface translocation is altered in HFD neutrophils.

In the light of the fact that neither GLUT1 nor 2-DG inhibited LPS-induced NET release by HFD neutrophils, it is interesting to note that PPP which is parallel to glycolysis and branches out of it once glucose-6-phosphate is formed, was involved in NET formation by HFD cells. However, only one (6-AN) of the two used inhibitors (DHEA) of PPP affected NET formation. The two act differentially, being competitive (6-AN) [85] and non-competitive inhibitors (DHEA) [86], which might explain the discrepancy. These prima facie unexpected results can be explained by the fact that neutrophils of obese mice derive from the inflammatory environment, as obesity is accompanied by low grade inflammation [7], whereas inflammatory neutrophils accumulate even 10-fold more glycogen than the noninflammatory/quiescent ones [87]. Thus, neutrophils of HFD mice might were utilizing glycogen for ATP production as inflammatory neutrophils do [18]. Consistently, inhibition of glycogen breakdown in neutrophils (either LPS-stimulated or nonactivated) reduces their survival in glucose-deprived conditions [18]. This implies that intracellular glucose stores are essential to meet neutrophil energy demands in conditions where fuels are limited, e.g., inflammation. Thus, we hypothesize that neutrophils from obese mice utilize intracellular, stored glucose which after the first step of glycolysis, branches toward the PPP pathway and fuels NETs release.

Whereas inhibition of the TCA cycle did not impact NET formation by any neutrophils studied here, some inhibitors of ATP synthase (complex V, FoF1 ATPase, fifth complex involved in oxidative phosphorylation) had an unexpected effect, both on ND and HFD cells. Namely, upon their application neutrophils were casting even more NETs. Oligomycin and Bz-423 are inhibitors of mitochondrial ATP synthase, but they operate via different mechanisms, namely Bz-423 (which binds to the oligomycin sensitivity conferring protein (OSCP)) impacts both V_max_ and Km of ATP synthase, whereas oligomycin (which binds to the c-ring of ATP synthases and blocks proton transport in the Fo subunit) affects exclusively V_max_ [88]. In contrast, angiostatin complexes with FoF1 ATPase (ATP synthase-β subunit) and inhibits mitochondrial activation [89]. Moreover, angiostatin serves as a ligand for surface ATPases and the majority of neutrophils do express surface/ectopic ATP synthase [90]. Surface ATP synthase may supply extracellular ATP (which may act though the purinergic receptor) and by pumping out protons it prevents intracellular acidosis [91]. Metabolic interplay between glycolysis and mitochondrial oxidation may lie at the root of the observed changes in neutrophils with blocked ATPases in regard to NET release. We hypothesize that cells with abolished mitochondrial metabolism compensate ATP production by increasing glycolysis, which results in stronger NET release. As glycolysis is the main ATP source in neutrophils, this process may be intensified to the maximum immediately upon LPS stimulation.

Enhanced glycolysis is observed during (auto)inflammatory disorders [92], and in diabetic individuals in whom expression of genes/proteins involved in glycolysis and PPP pathway is substantially upregulated in contrast to those engaged in mitochondrial metabolism (OXPHOS) [93]. On the other hand, stimulation with LPS of macrophages augments glycolysis, whereas oxygen consumption by the mitochondria is decreased [94]. Moreover, in dendritic cells, glycolytic metabolism is associated with nitric oxide (NO) production, and NO directly inhibits OXPHOS [95]. Neutrophils are plastic cells, not only in regard to the transcriptional profile of genes involved in their functioning during inflammation [96], but also in metabolic reprogramming to adapt metabolism to different environmental conditions [97]. Plasticity in switching between glycolysis and OXPHOS is also prominent during neutrophil differentiation [97]. On the other hand, PMA-induced NETs were hardly modulated by oligomycin [30]. Importantly, inhibition of mitochondrial ATP synthase with oligomycin had no effect on human neutrophil ATP levels, suggesting that compensatory mechanisms are triggered [16]. Increased release of NETs by neutrophils pretreated with oligomycin was previously described. The cells were activated with alum, and it was proposed to result from proton accumulation leading to the hyperpolarization of the inner mitochondrial membrane, and an increase in mitochondrial ROS production inducing NET release [98].

The major results on the capacity/efficiency to form NETs ex vivo were also confronted with actual NET release in the vasculature of ND and HFD mice during in vivo LPS challenge (6 h) or in intact animals (spontaneous NETs). The data hardly matches, showing that both intrinsic features of the cells and the milieu in which they function shape the overall process(es). Furthermore, the contrasting data show how challenging is to translate ex vivo data on NETs into the in vivo setting, as the former might be profoundly misleading. Nevertheless, these are only the studies on isolated neutrophils that allow for in depth studies on metabolic processes. This is because no sequestered metabolic manipulations are possible in vivo; for example, application of 2-DG into animals induces metabolic stress and leads to an enhanced cytokine release during endotoxemia [99]. But such a treatment affects all cells utilizing glycolysis and no cell-selective inhibition is possible. Thus, only some conditional genetic manipulations could facilitate the in vivo studies, e.g., induction of expression of Alternative oxidase (AOX) to free the excess of electrons in the mouse mitochondria, preventing therein ROS production [94]. Therefore, at the level of a single cell population it seems to be inevitable to first employ ex vivo studies.

**Figure 10:**
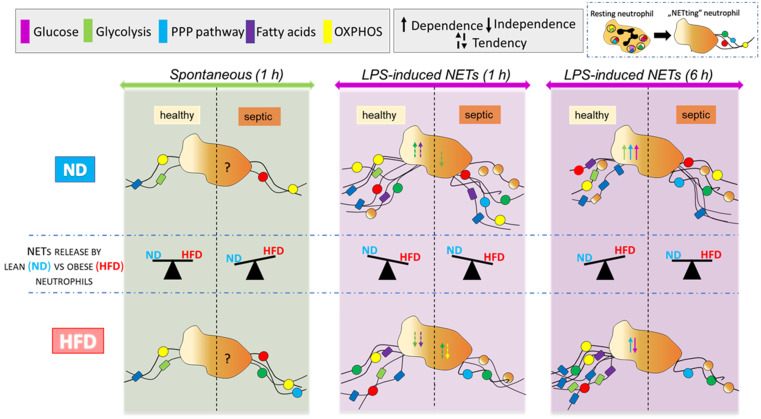
Key metabolic pathways/fuels regulating neutrophil extracellular trap (NET) formation by neutrophils of healthy and septic mice with obesity induced by high fat diet (HFD) and their lean controls (ND). Dependency/independency (and tendency) for glucose- (violet), glycolysis- (green), fatty acids- (navy blue), pentose phosphate pathway- (PPP, blue), oxidative phosphorylation-involvement (OXPHOS, yellow) in NET formation were marked with arrows in the corresponding colors. Short-term spontaneous (light green panel) and lipopolisaccharide (LPS)-induced (1 h, pink panel) NETs release was studied as well as long-term (6 h, pink panel) LPS-induced NETs were compared differences between them are presented as balanced/unbalanced scales.

In conclusion, with the application of the Seahorse technology and broad range of metabolic pathways inhibitors, we revealed that the requirements for fuels and pathways leading to NET formation differ between neutrophils originating from lean and obese individuals. In Figure 10, we present a compilation of results concerning main metabolic requirements for NET formation in obese and lean mice in all studied conditions. In particular, we revealed that obesity predisposes neutrophils from septic mice to spontaneously cast NETs driven by glycolysis and/or PPP pathway, and also possibly fatty acid oxidation. Even though neutrophils from obese septic subjects display high potential to utilize glycolysis they do not rely on it after the second hit of LPS (ex vivo) as they seem to be “exhausted” and hardly release NETs. In contrast, neutrophils of mice with regular body weight are highly glycolytic and maintain the ability to release NETs during systemic inflammation utilizing glucose and PPP pathways, independently or together. Putatively, the observed differences might be also linked to existence of different neutrophil subtypes in lean and obese mice. Additionally, our study revealed that mitochondrial ATP generation has an impact on NET release independently of adiposity, and this results from a cross-talk between metabolic pathways.

## 4. Materials and Methods

### 4.1. Mice

Animal studies were conducted under a protocol approved by the Local Ethical Committee No. II in Kraków (293/2017) and were in compliance with the EU Animal Care Guidelines. Three weeks old wild type C57BL/6J male mice were purchased from Charles River Laboratories (Sulzfeld, Germany; via AnimaLab) and placed either on a control diet (=normal diet, ND; Altromin, C1000, Fat 13%, Carbohydrates 67%, Protein 20% of kcal) or a high-fat diet (HFD; Altromin, C1090—60, Fat 60%, Carbohydrates 24%, Protein 16% of kcal) for at least 12 weeks. All the animals were weighed once a week, and throughout the feeding procedure, HFD-fed mice significantly increased their body mass in comparison to animals from the control diet group. Data on changes in weight, organs, and metabolic parameters of HFD mice were published previously [14]. Mice were housed under standardized conditions of temperature (21–22 °C) and illumination (12:12 h light/dark cycle) with food and tap water available ad libitum.

### 4.2. Induction of Systemic Inflammation/Endotoxemia

Systemic inflammation/endotoxemia was induced by an intraperitoneal (i.p.) injection of LPS (1 mg/kg body weight; *Escherichia coli* serotype 0111:B4; Sigma–Aldrich, Saint Louis, MO, USA) [14,100], and bone marrow (BM) neutrophils were harvested at 24 h post LPS challenge. In separate experiments, some animals were subjected to intravital imaging at 6 h of endotoxemia. In all experiments also LPS-untreated mice were used (designated as healthy mice).

### 4.3. Neutrophil Isolation

BM neutrophils were isolated from the femurs and tibiae. Mice were anesthetized with the mixture of ketamine hydrochloride (200 mg/kg b.w.; Biowet Pulawy, Pulawy, Poland) and xylazine hydrochloride (10 mg/kg b.w.; aniMedica, Südfeld, Germany) and then cervical dislocation was performed. Femurs and tibiae were separated from the rest of the body. The ends of the bones were cut off and the bone marrow was flushed with ice-cold HBSS(–) (w/o Ca^2+^ and Mg^2+^; Lonza Bioscience, Basel, Switzerland) using 25G needle. The bone marrow was then disintegrated by drawing it though a 20G needle. BM cells were pelleted by centrifugation (1300 rpm, 4 °C, 6 min) and then resuspended in 5 mL of 0.2% NaCl for about 30 s for hypotonic lysis of erythrocytes and osmolarity was immediately restored with 5 mL of 1.6% NaCl. Following centrifugation (1400 rpm, 4 °C, 7 min) the cell suspension was layered over a discontinuous Percoll gradient (GE Healthcare, Arlington Heights, IL, USA) consisting of 78, 69, and 52% Percoll solutions (2 mL of each) and subsequently spun at 2,600 rpm, 4 °C, for 30 min. Neutrophils localized between the 78 and 69% layers were collected and washed in HBSS(–) (1500 rpm, 4 °C, 6 min). Neutrophil pellet was suspended in HBSS(+) (w/Ca^2+^ and Mg^2+^, Lonza Bioscience, Basel, Switzerland) with normal glucose level (NG; 5.5 mM) or HBSS(+) supplemented with high glucose concentration (HG; 22 mM) or in PBS (Gibco, Waltham, MA, USA). D-mannitol (16.5 mM; Sigma–Aldrich, Saint Louis, MO, USA) was used as an osmotic control for high glucose conditions.

Neutrophils were stained with Trypan blue dye to estimate their viability (Sigma–Aldrich, Saint Louis, MO, USA) and the neutrophil counts and purity were determined by Türk solution staining (0.01% crystal violet in 3% acetic acid; Sigma–Aldrich, Saint Louis, MO, USA) upon cell counting in a hemocytometer. The purity of isolated neutrophils was ~99% as estimated by their morphology (Türk/hemocytometer) and ~70% according to flow cytometry (Ly6G^+^ cells), and neutrophil viability was ~98% (Trypan blue) in each experiment. Less than 50% of neutrophils was retrievable from BM of septic vs. healthy mice, independently of their origin from ND or HFD mice. This is because neutrophils are mobilized from bone marrow during sepsis [101].

### 4.4. Metabolic Pathway Inhibitors

Bone marrow neutrophils were incubated with metabolic pathway inhibitors for 1 h before lipopolysaccharide stimulation (75 μg/mL, final well conc.) for next 6 h. Glycolysis was inhibited with 2-deoxy-D-glucose (2-DG, 20 mM; Sigma–Aldrich, Saint Louis, MO, USA). Pentose phosphate pathway (PPP) was inhibited using 6-aminonicotinamide (6-AN, 5 µM; Sigma–Aldrich, Saint Louis, MO, USA) and dehydropiandrosterone (DHEA, 100 μM; Sigma–Aldrich, Saint Louis, MO, USA). Dimethyl malonate (DMM, 10 mM; Sigma–Aldrich, Saint Louis, MO, USA) was used to inhibit Krebs cycle (tricarboxylic acid cycle, TCA cycle) and range of inhibitors were applied to inhibit oxidative phosphorylation (OXPHOS): rotenone (2 μg/mL, complex I inhibition; Sigma–Aldrich, Saint Louis, MO, USA), antymycin A (1 μM, complex III inhibition; Sigma–Aldrich, Saint Louis, MO, USA), mitochondrial ATP synthase inhibitors; oligomycin (5 μg/mL; Sigma-Aldrich, Saint Louis, MO, USA), piceatannol (50 μM; Sigma–Aldrich, Saint Louis, MO, USA), Bz-423 (10 μM; Tocris Bioscience, Bristol, UK). Surface ATPase was blocked with angiostatin K1-3 human recombinant (6 μg/mL; BioVision, Milpitas, CA, USA). Additionally, glucose transporter, GLUT1 was inhibited with WZB117 (10 μM; Sigma–Aldrich, Saint Louis, MO, USA).

### 4.5. Quantification of NETs by Fluorescence Spectrophotometry

Extracellular DNA was stained with 5 μM SYTOX green (Invitrogen, Carlsbad, CA, USA), a fluorescent membrane-impermeable DNA dye. Fluorescence was quantified using a microplate reader at 485/535 nm (Tecan, Infinity F200 Pro, Männedorf, Switzerland).

### 4.6. Quantification and Visualization of NETs by Fluorescence/Confocal Microscopy

Neutrophils (5×10^4^ cells/mL) were seeded on noncoated glass slides (ᴓ5 mm; ThermoScientific, Waltham, MA, USA) and allowed to adhere for 30 min at 37 °C with 5% CO_2_. Thereafter, they were incubated with metabolic pathways inhibitors for 1 h as described above and/or stimulated for 6 h with LPS (75 μg/mL, final well concentration) [14,102]. After incubation, cells were fixed with increasing concentrations of paraformaldehyde solution (PFA, 1, 2, 3% in PBS for 2, 10, 20 min, respectively) and extracellular DNA was stained with 5 μM SYTOX green (Invitrogen, Carlsbad, CA, USA). Lastly, coverglasses were mounted with VECTASHIELD Mounting Medium (Vector Laboratories, Burlingame, CA, USA). Alternatively, immunostaining of neutrophil elastase (NE, clone EPRendothelial79) or citrullinated histone H3 (citH3) was performed. Briefly, fixed cells were washed for 5 min in PBS, and then incubated in blocking solution (3% BSA in PBS) (BSA, bovine serum albumin; Sigma–Aldrich, Saint Louis, MO, USA) for 45 min at RT. Subsequently, incubation with rabbit monoclonal anti-NE antibody (Abcam, Cambridge, UK) or rabbit polyclonal anti-histone H3 (citrulline R2 + R8 + R17) antibody, both diluted 1:200 in 1% BSA/PBS (Abcam, Cambridge, UK), was performed (4 °C, overnight in a humid chamber). The slides were then washed two times in PBS (5 min) and incubated with Cy3-conjugated goat anti-rabbit IgG (H+L) antibody (diluted 1:300 in PBS/1% BSA; Jackson Immunoresearch, Ely, UK) for 1 h at RT. Finally, 5 μM SYTOX green was added to stain for extDNA. After washing in PBS, the coverglasses were mounted with VECTASHIELD Mounting Medium (Vector Laboratories, Burlingame, CA, USA).

NETs were visualized using the ZEISS Axio Vert. A1 inverted microscope with ZEN 2.3.SP1 software (Carl Zeiss Ltd, Cambridge, UK) and ZEISS Axio Examiner.Z1 upright microscope equipped with a metal halide light source (AMH-200-F6S; Andor, Oxford Instruments, Abingdon, UK) with motorized 6 position excitation filter wheel and laser-free confocal spinning disk device (DSD2; Andor, Oxford Instruments, Abingdon, UK) with ZEISS EC Plan-NEOFLUAR 20×/0.5 and 40×/0.6 air objective. Two excitation filters (GFP: 482/18 nm; RFP: 561/14 nm) and appropriate emission filters (GFP: 525/45 nm, RFP: 609/54 nm) were used. The 5.5 megapixel sCMOS camera (Zyla 5.5; Andor, Oxford Instruments, Abingdon, UK) was applied for fluorescence detection. An iQ 3.6.1 acquisition software (Andor, Oxford Instruments, Abingdon, UK) was used to drive the microscope. Images were analyzed with ImageJ v1.53a software as previously described [14]. Briefly, images were changed to a grayscale (8-bit type) and thresholded to assess the signal from extDNA/NET which eventually was expressed as percentage of FOV covered area.

### 4.7. Glucose Transporter (GLUT1) Immunostaining

Prior to staining, neutrophils seeded on coverglasses were permeabilized by bathing in TBS (Triton X-100, Na_2_HPO_4_ × 12H_2_O, Na_2_HPO_4_ × 1H_2_O, BSA, NaCl, dH_2_O) for 5 min. Nonspecific antibody binding was blocked by incubation with 3% BSA/PBS for 45 min in RT. Subsequently, the cells were labeled with recombinant rabbit monoclonal antibody anti-Glucose Transporter GLUT1 (clone EPR3915; Abcam, Cambridge, UK; diluted 1:100) and incubated overnight at 4 °C in a humid chamber. The slides were then washed 2 times in PBS and incubated with the secondary antibody goat anti-rabbit IgG–H&L (Cy3) (diluted 1:100) for 1 h at RT. After the coverglasses were washed in PBS, SYTOX green was added to stain for DNA/nuclei (5 μM) and slides were mounted with VECTASHIELD Mounting Medium. Fluorescent signal was detected with a ZEISS Axio Examiner.Z1 upright microscope equipped with confocal spinning disk device DSD2 as described above.

### 4.8. Mitochondrial Tracker Green and Janus Green B Staining

To label mitochondria, neutrophils were seeded in 96-well plates (Nest Scientific, Rahway, NJ, USA) and let to adhere for 30 min. After that time, cells were incubated with MitoTracker^®^green FM (200 nM final conc.; ThermoFisher Scientific, Waltham, MA, USA), which passively diffuses across the plasma membrane and accumulates in active mitochondria. After 45 min incubation, cells were pelleted by centrifugation (1500 rpm, RT, 5 min) and resuspended in fresh prewarmed HBSS(+). Cells were analyzed by fluorescence microscopy (ZEISS Axio Vert. A1 inverted microscope with ZEN 2.3.SP1 software, Carl Zeiss Ltd, Cambridge, UK) and spectrophotometrically using a microplate reader at 485/535 nm (Tecan, Infinity F200 Pro, Männedorf, Switzerland). The reduction of Janus green B was studied by adding Janus Green B solution (0.1% in phosphate buffer; Pol-Aura, Zabrze, Poland) to cell suspension at 1:1 ratio and analyzed with light microscopy (ZEISS, Primo Star, Cambridge, UK).

### 4.9. Seahorse Analysis

The cellular bioenergetics were determined using the XFp analyser (Agilent, Boston, MA, USA) kindly provided by Perlan Technologies Poland. All assays were programmed (designed) in XF data acquisition Wave 2.6.1 software (Agilent, Boston, MA, USA). In each experiment, 3 baseline measurements were taken prior to the addition of any compound/substrate/inhibitor, and at least 3 response measurements were taken after the addition of each compound. Oxygen Consumption Rate (OCR) and Extracellular Acidification Rate (ECAR) were reported as absolute rates (pmoles/min for OCR and mpH/min for ECAR). While sensor cartridges were hydrated (overnight) and calibrated (XF Calibrant), cell plates were incubated in a 37 °C for 30 min prior to the start of an assay. All experiments were performed at 37 °C in non-CO_2_ conditions. Detailed protocols and their justification can be found at https://www.agilent.com/en/product/cell-analysis/how-to-run-an-assay (accessed from June 2020 to May 2021). Additionally, detailed protocols were previously published [103,104].

#### 4.9.1. Seahorse XF Measurement of ECAR and OCR

Neutrophils were suspended in sterile (0.2 um syringe strainer filtered) HBSS(+) w/o sodium bicarbonate (Gibco, Waltham, MA, USA) supplemented with 1 mM sodium pyruvate (Sigma–Aldrich, Saint Louis, MO, USA), 2 mM L-Glutamine (Sigma–Aldrich, Saint Louis, MO, USA), 10 mM D-glucose (Lonza Bioscience, Basel, Switzerland) and 5 mM HEPES (Sigma–Aldrich, Saint Louis, MO, USA) and adjusted to pH 7.4 with 0.1 N NaOH (Sigma–Aldrich, Saint Louis, MO, USA). Buffer factor of assay media was validated prior to experiments and was equal to 2,9 mM/pH. Cells were plated (300,000 cells/well) in 180 μL on Agilent Seahorse 8-well XFp Cell Culture Miniplate and allowed to settle/adhere for 30 min at 37 °C. Real-time, noninvasive measurements of ECAR and OCR were obtained which correlated to acidification, mostly derived from glycolysis and mitochondrial function, respectively. While the assay was running, at 12 min of baseline measurement, acute injection of LPS suspended in assay medium (75 µg/mL final well conc., port A, 20 μL) was done, and measurements were continued for 1 h. Measurements taken after the LPS injection consisted of (i) a sample mixing time (each 1 min long) and (ii) a data acquisition period of 57 min. The latter consisted of 3 cycles with waiting time before each measurement lasting for 15 min.

#### 4.9.2. Seahorse XF Glycolysis Stress Test Assay

Neutrophils were suspended in PBS (glycolysis stress test medium, w/o glucose and pyruvate supplementation, pH 7.4), plated (300,000 cells/well) in 160 μL on Agilent Seahorse 8-well XFp Cell Culture Miniplate and allowed to adhere for 30 min at 37 °C. Just before running the assay, neutrophils were stimulated with LPS (75 µg/mL final well conc.) and immediately placed in the instrument. The assay workflow was as follows: (1) injection of glucose (10 mM; rate of glycolysis), (2) injection of ATP synthase inhibitor - oligomycin (1 µM; cellular maximum glycolytic capacity). (3) Lastly, 2-DG (50 mM) was injected to abolish glycolysis. Measurements after each injection consisted of a mixing time of 3 min each, and a data acquisition period of 18 min consisting of 3 cycles. The difference between glycolytic capacity and glycolysis rate defines glycolytic reserve. Three cycles of baseline measurement (total duration of 18 min) were taken prior to the addition of glucose. ECAR, prior to glucose injection, is referred to as nonglycolytic acidification. All parameters were calculated using Agilent Seahorse XF Glycolysis Stress Test Report Generator.

#### 4.9.3. Seahorse XF Mito Fuel Flex Test

Neutrophils were suspended in sterile/filtered HBSS(+) w/o sodium bicarbonate (Gibco, Waltham, MA, USA) supplemented with 1 mM sodium pyruvate (Sigma–Aldrich, USA), 2 mM L-Glutamine (Sigma–Aldrich, Saint Louis, MO, USA), 10 mM D-glucose (Lonza Bioscience, Basel, Switzerland, USA), 5 mM HEPES (Sigma-Aldrich, Saint Louis, MO, USA), adjusted to pH 7.4 with 0.1 N NaOH (Sigma–Aldrich, Saint Louis, MO, USA). Cells were plated (300,000 cells/well) in 160 μL on Agilent Seahorse 8-well XFp Cell Culture Miniplate. Just before running the assay, neutrophils were stimulated with LPS (75 µg/mL final well conc.) and immediately placed in the instrument. Following inhibitors required to determine the dependency, capacity, and flexibility of cells for long chain fatty acids oxidation were used: Etomoxir (40 μM, final well conc., port A, 20 µL; Sigma–Aldrich, Saint Louis, MO, USA)—an inhibitor of long chain fatty acid oxidation, UK5099 (20 μM, final well conc., port B, 22 µL, Sigma–Aldrich, Saint Louis, MO, USA)—an inhibitor of the glucose oxidation, BPTES (30 μM, final well conc., port C, 25 µL, Sigma–Aldrich, Saint Louis, MO, USA)—an inhibitor of the glutamine oxidation. Three cycles of baseline measurements (57 min) with a mixing time (1 min) and waiting time before each cycle (15 min) were taken. Subsequently, measurements after each inhibitor were performed. They consisted of a mixing time of 3 min and a data acquisition period of 30 min consisting of 3 cycles. Fatty acid (FA) dependency was tested by first injecting Etomoxir, followed by inhibition of the two alternative pathways (UK5099/BPTES). FA capacity was tested by first injecting UK5099/BPTES, followed by injection of Etomoxir. Fuel Flexibility was calculated as the difference between capacity and dependency. All parameters were calculated using Agilent Seahorse XF Mito Fuel Flex Test Report Generator.

### 4.10. Staining of NETs In Vivo with Intravital Microscopy

Mice were anesthetized with a mixture of ketamine/xylazine as described above, and then cannulation of the right jugular vein was performed. Preparation of the liver for intravital imaging was performed as previously described by Kolaczkowska et al. [51]. Spinning-disk confocal intravital microscopy was performed using ZEISS Axio Examiner.Z1 upright microscope equipped with confocal spinning disk device DSD2 (described above). The following filters were used: four excitation filters (DAPI: 390/40 nm; GFP: 482/18 nm; RFP: 561/14 nm; Cy5: 640/14 nm) and appropriate emission filters (DAPI: 452/45 nm, exposure time 600 ms; GFP: 525/45 nm, exposure time 700 ms; RFP: 609/54 nm, exposure time 500 ms; Cy5: 676/29 nm, exposure time 900 ms). Following antibodies were used to detect presence of NETs and neutrophils in the liver vasculature: Alexa Fluor 647 antineutrophil elastase (1.6μg/mouse, clone G-2; Santa Cruz Biotechnology, Dallas, TX, USA), histone H2A.X (0.5µg/mouse, clone 938CT5.1.1; Santa Cruz Biotechnology, Dallas, TX, USA), Brilliant Violet 421 anti-Ly6G (1.6 μg/mouse, clone 1A8; BioLegend, San Diego, CA, USA). All antibodies were injected intravenously (i.v.) via the jugular vein ~20 min prior to intravital imaging as previously published methodology [14].

### 4.11. Statistics

All data are presented as mean values ± SD. Data were compared by unpaired two-tailed Student’s t test and one way analysis of variance (ANOVA) with Bonferroni multiple comparisons post hoc test (ANOVA: any two means that do not share the same letter are significantly different). Statistical significance was set at *p* < 0.05. The experiments were repeated 2–3 times with n ≥ 3 per group.

## 5. Conclusions

We conclude that metabolic alterations present in obese mice impact neutrophil functioning and dependency on fuels. They are especially apparent shortly after cell isolation and normalize with time. However, they reflect on neutrophil abilities to produce NETs during the inflammatory response in different metabolic milieu. Therefore, modulation of metabolic processes may offer a possibility to regulate release of NETs during life-threatening infections.

## Data Availability

All data is contained within the manuscript.

## Supplementary Materials

The supplementary material are available online at https://www.mdpi.com/article/10.3390/ijms22147718/s1.
