# Supplementary material for: Metabolic Pathways Involved in Formation of Spontaneous and Lipopolysaccharide-Induced Neutrophil Extracellular Traps (NETs) Differ in Obesity and Systemic Inflammation
Source: Int J Mol Sci. 2021 Jul 19;22(14):7718. doi: 10.3390/ijms22147718 (PMC8303382; doi:10.3390/ijms22147718)
Supplement: Supplementary file 1 [file ijms-22-07718-s001.zip › ijms-1251580-supplementary.pdf]

Supplementary Figures

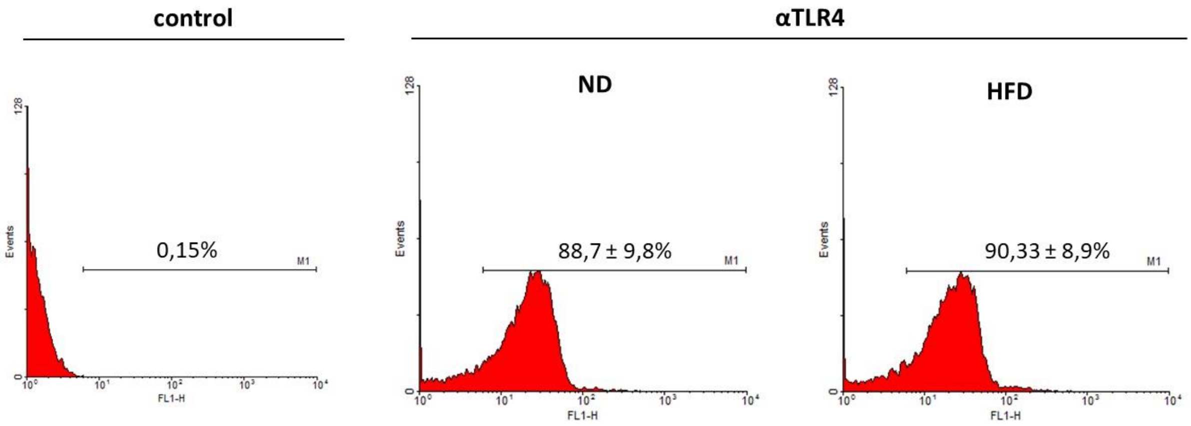

Supplementary Figure S1. Flow cytometric analysis of TLR4 expression on neutrophils. Representative histograms show TLR4 expression on neutrophils of lean (ND) and obese (HFD) mice (right panel). Control cells are shown for lean mice on the left histogram. Following antibodies were used—unconjugated rat anti-mouse TLR4/MD-2 Complex antibody (BD Bioscience), Alexa Fluor® 488 AffiniPure anti-rat IgG antibody (Jackson Immunoresearch) (FL-1). Positive cell quantification is given from two repetitions. The number of TLR4+ cells in each group is expressed as a % ± s.d (n=2).
